# E-health based solutions for improving quality of antibiotic use in hospitals

**DOI:** 10.1186/1753-6561-5-S6-P36

**Published:** 2011-06-29

**Authors:** CA Palos, C Santos, JA Gomes, M Ressurreição, C Alves

**Affiliations:** 1 Hospital Da Luz, Portugal; 2 Siemens Medical Solutions Portugal, Lisboa, Portugal

## Introduction / objectives

Multidrug resistant microorganisms (MDROs) constitute a serious threat. Strategies to reduce incidence are based on reducing both emergence and transmission. e-health, defined as intensive use of information and communication technologies, can be a major part of strategies to reduce the rate of MDROs.

## Methods

Aiming improvement of the quality of antibiotic use based on the e-health concept in a 280-bed, paper-free, general hospital, Infection Control Committee (ICC), along with Quality and Antibiotics Committees (Q&A) and IT team, has been working on implementation of new tools at several levels. On the electronic medical record, a new template for antimicrobial prescription has been created and is in use since January, conditioning automatically the prescription in terms of context (surgical antibiotic prophylaxis in accordance with type of surgery; medical therapy, in accordance with the type of infection) and duration (intra-operative, 1-day or 2-days for antibiotic surgical prophylaxis; 7 days maximum for therapeutics). If there is a disagreement between prescriptions and protocols, physicians may proceed, but an electronic justification must be fulfilled (which is also obligatory for some antimicrobials) and sent to the ICC and Q&A Committees, which, in turn interact with prescribers both by e-mail and phone call, thus building up an antibiotic stewardship. This is complemented by e-pub of antimicrobial protocols on the hospital intranet and by release of data on antibiotic use.

## Results

Data analysis is still on process.

## Conclusion

The authors hope that the use of e-health on antibiotic management will improve the quality of care and thus reducing MDROs emergence.

## Disclosure of interest

None declared.

